# Insights Into Translatomics in the Nervous System

**DOI:** 10.3389/fgene.2020.599548

**Published:** 2020-12-21

**Authors:** Shuxia Zhang, Yeru Chen, Yongjie Wang, Piao Zhang, Gang Chen, Youfa Zhou

**Affiliations:** ^1^Department of Anesthesiology, Sir Run Run Shaw Hospital, School of Medicine, Zhejiang University, Hangzhou, China; ^2^Key Laboratory of Elemene Anti-Cancer Medicine of Zhejiang Province and Holistic Integrative Pharmacy Institutes, Hangzhou Normal University, Hangzhou, China; ^3^Engineering Laboratory of Development and Application of Traditional Chinese Medicine from Zhejiang Province, Holistic Integrative Pharmacy Institutes, Hangzhou Normal University, Hangzhou, China

**Keywords:** translatomics, RNC-mRNA, the nervous system, polysome profiling, ribosome profiling, translating ribosome affinity purification

## Abstract

Most neurological disorders are caused by abnormal gene translation. Generally, dysregulation of elements involved in the translational process disrupts homeostasis in neurons and neuroglia. Better understanding of how the gene translation process occurs requires detailed analysis of transcriptomic and proteomic profile data. However, a lack of strictly direct correlations between mRNA and protein levels limits translational investigation by combining transcriptomic and proteomic profiling. The much better correlation between proteins and translated mRNAs than total mRNAs in abundance and insufficiently sensitive proteomics approach promote the requirement of advances in translatomics technology. Translatomics which capture and sequence the mRNAs associated with ribosomes has been effective in identifying translational changes by genetics or projections, ribosome stalling, local translation, and transcript isoforms in the nervous system. Here, we place emphasis on the main three translatomics methods currently used to profile mRNAs attached to ribosome-nascent chain complex (RNC-mRNA). Their prominent applications in neurological diseases including glioma, neuropathic pain, depression, fragile X syndrome (FXS), neurodegenerative disorders are outlined. The content reviewed here expands our understanding on the contributions of aberrant translation to neurological disease development.

## Introduction

Gene expression is a cellular process involving the transcription, post-transcription modification, translation, messenger RNAs degradation, and protein turnover (Liu et al., 2016). Each step of this cascade shapes and balances gene expression. How this balance is achieved and the extent to which these processes contribute to protein synthesis is a long-standing open question. Although transcriptomic and proteomic techniques have greatly improved our ability to understand gene expression changes, significance is limited by a lack of strictly direct correlation between mRNA and protein levels, with some abundant mRNAs being poorly translated and vice versa. However, protein levels correlate better with mRNAs attached to ribosome-nascent chain complex (RNC-mRNA) than with total mRNA levels, attaining a correlation of about 0.95 (Gygi et al., 1999; Greenbaum et al., 2003; Wang et al., 2013). Gene expression patterns derived from transcriptomic analysis corresponding to steady-state mRNA levels do not take translational control into consideration, which accounts for a large part of all regulatory amplitudes. Collectively, these observations highlight the importance of translational control. Indeed, various neurological diseases, including neuropathic pain (Colloca et al., 2017), neurodegenerative disorder (Poewe et al., 2017), multiple sclerosis (Filippi et al., 2018), fragile X syndrome (FXS) (Hagerman et al., 2017) depression (Aguilar-Valles et al., 2018) result from aberrant translation. Multiple studies have evaluated gene expression changes caused by external stimuli (Floriou-Servou et al., 2018) and neurological disorders (Shawahna et al., 2011; Donega et al., 2019) combining transcriptomic and proteomic profiling. However, their resolution is limited by high cellular heterogeneity (Sharma et al., 2015), alternative splicing (Su et al., 2018), translational regulation (Shigeoka et al., 2016), protein degradation (Daniele et al., 2018), and protein localization (Holt et al., 2019) likely causing the poor correlation seen between mRNAs and protein levels in the nervous system. Besides, insufficient sensitivity by proteomics limits detection of small amounts of newly synthesized protein that may accumulate in extremely narrow time windows, given that proteins are more stable (median half-life of 46 h) than mRNAs (Schwanhäusser et al., 2011). In addition to translation, protein levels are also influenced by degradation, which is also tightly regulated. Consequently, it reinforces the requirement for RNA analysis at a point closest to the protein, then the translatome steps onto the stage to obtain more complete information of neurological development and dysfunction.

Polysome profiling, the first translatomics technique, has been valuable in translational studies – uncovering dynamics of ribosome occupancy and density. Ribosome profiling is another promising technique that can precisely determine mRNA localization on loaded ribosomes. Translating ribosome affinity purification (TRAP), which is widely used in nervous system studies, is suited for examining translation in specific cell types within complex brain tissues.

In this review, we discuss the generalized view of translatome, which covers almost all elements involved in translation, and how their dysregulation affects neurodevelopment. We then highlight how the three main translatomics methods identify mRNAs under translation and how they exert their functions in the neurological diseases. Next, the other reported applications of translatomics in the nervous system are concluded, which may lead to new ideas to study translational changes of neurological diseases. Finally, based on its application in other fields, we speculate on future translatomics applications in neuroscience.

## Broad Sense Translatomics

**FIGURE 1:**
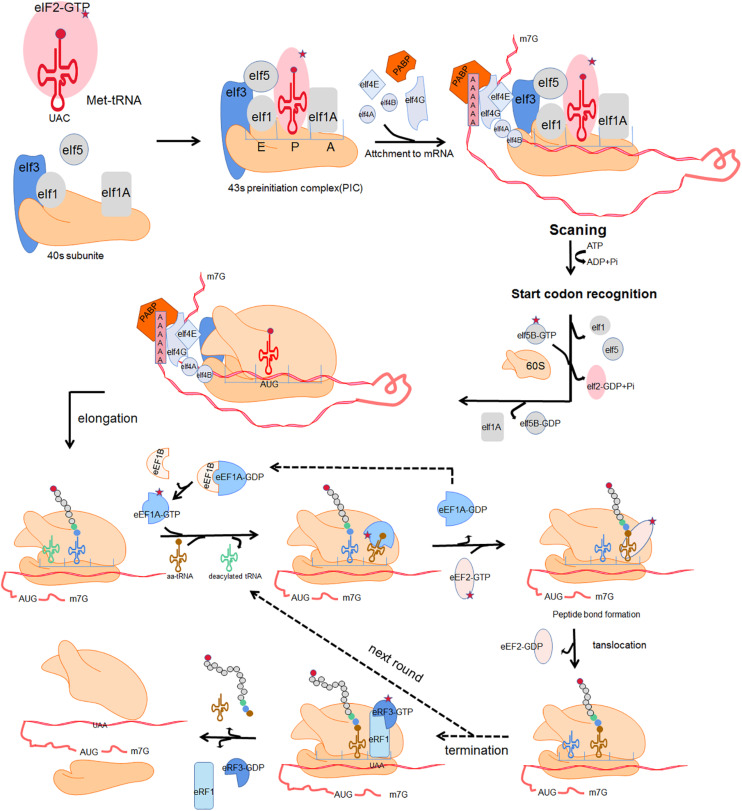
Four fundamental phases of translation:initiation, elongation, termination and ribosome recycle. Initiation: a ternary complex (TC) comprising a GTP-bound eukaryotic translation initiation factor 2 (eIF2) and methionyl-tRNA (Met-tRNA_*i*_^*Met*^) interacts with the 40s ribosomal subunit and several initiation factors including eukaryotic translation initiation factor 1 (eIF1), eukaryotic translation initiation factor 1A (eIF1A), eukaryotic translation initiation factor 5 (eIF5) and eukaryotic translation initiation factor 3 (eIF3), forming an 43s preinitiation complex (43s PIC). The 43s PIC is recruited to the mRNA 5′ cap through the eukaryotic translation initiation factor 4B (eIF4B) and eukaryotic translation initiation factor 4F (eIF4F) which is composed of eukaryotic translation initiation factor 4A (eIF4A), eukaryotic translation initiation factor 4E (eIF4E) and eukaryotic translation initiation factor 4G (eIF4G). Poly(A)-binding protein (PABP) together with eIF4G acts as a bridging factor which mediates “closed loop” mRNA conformation. The 43s PIC then scans along the mRNA until the P-site-bound Met-tRNA with anticodon is correctly paired with the mRNA start codon. Subsequently the dissociation of eIF2 and other eukaryotic initiation factors trigger the combination of the 60S and the 40S ribosomal subunit, forming a complete 80s ribosome and allowing the second codon to be decoded at the A site. Elongation: the incoming aminoacyl-tRNA (aa-tRNA) with the complementary codon enters the ribosome A site, facilitated by eukaryotic translation elongation factor 1A (eEF1A) and eukaryotic translation elongation factor 1B (eEF1B). A peptide bond is established between the incoming amino acid of the A site and the adjacent amino acid of peptidyl-tRNA in the ribosomal P site. Subsequently, the nascent peptide of the P site leaves its tRNA and binds to the amino group of the A-site aminoacyl-tRNA. Once a peptide bond is formed, the ribosome slides forward relative to the mRNA triggering translocation of the tRNAs into the canonical P- and E-sites. This is promoted by eukaryotic translation elongation factor 2 (eEF2) with transposase activity at the cost of GTP hydrolysis. The release of E-site deacylated tRNA occurs before the next aminoacyl-tRNA enters into the vacant A site. Termination and ribosome recycle: translation termination occurs when a stop codon enters the A site of ribosome. In eukaryotes, eukaryotic release factor 1 (eRF1) and eukaryotic release factor 3 (eRF3) hydrolyze the ester bond of the peptidyl-tRNA, driving the translation termination and ultimately triggering the release of nascent polypeptide chains. The ribosome dissociates into separate subunits and escapes from mRNA.

mRNA translation is the process by which the “base sequence” (nucleotide sequence) of mature mRNA molecules is used to synthesize corresponding specific amino-acid sequences. This highly dynamic process is divided into 4 crucial phases: initiation, elongation, termination, and ribosome recycling (Rodnina, 2018). In each phase, the ribosome and auxiliary translation factors form transient complexes that facilitate protein synthesis. Figure 1 shows a schematic representation of translation (Kapur et al., 2017). Initiation involves assembly of a ternary complex (TC) including the 40S ribosomal small subunit (SSU), the associated eukaryotic initiation factors (eIFs) and methionyl-tRNA (Met-tRNA_*i*_^*Met*^) near the mRNA 5′ cap; the SSU then scans along the mRNA in the 3′ direction until the P-site-bound Met-tRNA with anticodon is correctly paired with the mRNA start codon. the SSU is joined by the 60S ribosomal large subunit (LSU) to form the 80S ribosome. The incoming aminoacyl-tRNA (aa-tRNA) with the complementary codon enters the ribosome A site, and elongation commences. A peptide bond is established between the incoming amino acid of the A site and the adjacent amino acid of peptidyl-tRNA in the ribosomal P site. Once a peptide bond is formed, the ribosome slides forward relative to the mRNA triggering translocation of the tRNAs into the canonical P- and E-sites. Translation termination occurs when a stop codon enters the A site of ribosome. The nascent polypeptide chains release, then the ribosome dissociates into separate subunits and escapes from the mRNA.

The term translatome refers to the totality of elements directly involved in translation, including ribosomes, RNC-mRNA, tRNA, regulatory RNA (such as miRNA and lncRNA), RNA binding proteins, and various translation factors (Zhao et al., 2019). Impaired function by these elements is associated with a range of pathophysiological changes in the nervous system (Table 1).

**TABLE 1:** Elements involved in translation and the application of translatomics in nervous system diseases.

**Elements**	**Conclusions**		**References**
Ribosome	Dysregulation of ribosomal biogenesis		Zanni et al., 2015; Slomnicki et al., 2016, 2018
	disrupts neurodevelopment.		
tRNA	Interruption of tRNA maturation and modification		Karaca et al., 2014; Ivanova et al., 2017; Lin et al., 2018;
	drives nervous system dysfunctions.		Ramos and Fu, 2019; Schaffer et al., 2019; Torres et al., 2019
Auxiliary translation	Initiation factors (e.g., eIF4E and eIF2α) and elongation factors		Beckelman et al., 2016; Amorim et al., 2018;
factors	(e.g., eEF1A and eEF2) are involved in neurodevelopment		Jan et al., 2018; Tsai et al., 2018; Uttam et al., 2018b;
	and maintenance of synaptic plasticity.		Zyryanova et al., 2018
Regulatory RNAs	Inhibit translation, mediated by microRNAs decreasing protein levels.		Schratt et al., 2006; Murphy et al., 2017; Zhang H. et al., 2018; Chen et al., 2020
	IncRNAs and circRNAs function as competing endogenous RNAs		
	by sponging microRNAs in neuronal cells.		
RNA binding proteins	Some RNA binding proteins (e.g., FMRP and PUM2) repress		Chen et al., 2014; D’Amico et al., 2019
	the translation of their target mRNAs.		Chen et al., 2014; D’Amico et al., 2019
	Diseases	Conclusions	
RNC-mRNAs	Glioma	Polysome profiling gives the first insight into the radiation and drug resistance of GBM cells at the translation level.	Wahba et al., 2016, 2018; Bell et al., 2018
	Neuropathic Pain	Nav1.8-TRAP mice could achieve sensory neuron-specific ribosome tagging with enrichment in the nociceptor population and facilitate the finding of new mechanisms controlling nociceptor excitability. Axon-TRAP is likely lead to important breakthroughs in our understanding of which mRNAs are translated locally in nociceptor axons to modulate excitability.	Shigeoka et al., 2016; Megat et al., 2019a,b
	Depression	Astroglial-specific bacTRAP connects perineuronal net, astroglial cells, and depression. Similarly, ePet-Cre^*tg/*–^/RiboTag^*tg/*–^mice link FKBP5 and serotonin with depression.	Simard et al., 2018; Lesiak et al., 2020
	Fragile X syndrome	Ribosome profiling can distinguish stalled and active ribosomes, and uncovered the contribution of ribosomal brake -FMRP to fragile X syndrome.	Das Sharma et al., 2019; Shah et al., 2020
	Neurodegenerative diseases	TRAP method targeting cell types defined by genetics or projections can effectively profile translation regulation on memory consolidation associated with Alzheimer’s disease. Accurately interference with the translation mechanism in specific cells of targeted regions may be a novel therapeutic target for neurodegenerative diseases.	Cho et al., 2015; Ostroff et al., 2019; Voskuhl et al., 2019; Kim et al., 2020; Lim et al., 2020

Translation is executed by ribosomes (Peña et al., 2017), which are comprised of a 60S subunit that possesses 3 ribosomal RNAs (25S, 5.8S, 5S) and 47 proteins, and a 40S subunit containing 18S ribosomal RNA and 33 ribosomal proteins. Ribosomopathies are a diverse group of disorders associated with aberrant ribosome production and function (Mills and Green, 2017). Impaired expression of ribosomal genes in the brain causes ribosome biogenesis abnormalities in mice suffering from chronic social defeat stress (Smagin et al., 2016). Using X-exome resequencing, a mutation affecting RPL10, a 60S ribosomal protein, causes neurodevelopmental defects and X-linked disorders (Zanni et al., 2015; Slomnicki et al., 2016). Thus, dysregulation of ribosomal biogenesis and function may disrupt neurodevelopment, leading to microcephaly and cognitive impairment (Ingolia et al., 2009; Darnell, 2014; Ishimura et al., 2014; Zanni et al., 2015; Slomnicki et al., 2016). Using conventional biochemical methods, knockout or knockdown of ribosome proteins in neurons could mimic the observed impaired ribosomal biogenesis in neurodegenerative diseases and study their pathogenic contributions (Slomnicki et al., 2018). The structural biology method has also made remarkable contributions to the ribosome field. Our current understanding of ribosome structure and function has been greatly expanded after the emerging of X-ray crystallography and cryogenic electron microscopy (cryo-EM) (Wilson and Doudna Cate, 2012; von Loeffelholz et al., 2017). High-resolution features of cryo-EM has enabled the visualization of chemical modifications of the ribosomal RNA and the ligand complexes of the ribosome (Myasnikov et al., 2016; Javed et al., 2017; von Loeffelholz et al., 2017). Cryo-EM has been used to analyze the structure of receptors and ion channels in neurons (Dang et al., 2017; Laverty et al., 2019; Liu et al., 2019), but few studies have reported ribosomopathies under cryo-EM.

Disruption in tRNA maturation and modification can alter the amount of cellular tRNA, thereby damaging protein folding and affecting neuronal homeostasis, which may result in nervous system dysfunction (Blanco et al., 2014; Kapur et al., 2017; Musier-Forsyth, 2019). tRNA aminoacylation by tRNA synthetases is a critical quality control checkpoint for maintaining translation fidelity. Mutations in aminoacyl-tRNA synthetases are implicated in a wide range of nervous system disorders (Meyer-Schuman and Antonellis, 2017; Ognjenović and Simonović, 2018). Various factors have linked tRNA dysfunction to neurological disorders, including a genomic mutation that reduces the expression of isodecoder, which has the same anticodon as tRNA but differs in the tRNA body sequence (Torres et al., 2019), mutations affecting tRNA splicing machinery (Karaca et al., 2014; Ivanova et al., 2017), aberrant expression of tRNA metabolism genes (Schaffer et al., 2019), and impaired tRNA modification (Lin et al., 2018; Ramos and Fu, 2019) all point to an association between defective tRNA function and neurological disorders. Recently, DM-tRNA-seq (demethylase tRNA sequencing), modified charged DM-tRNA-seq and ARM-seq (AlkB-facilitated RNA methylation sequencing) have developed to identify and quantify tRNA base modification (Cozen et al., 2015; Zheng et al., 2015; Evans et al., 2017). These advances have not been applied to the neuronal field, but future applications of these technologies may provide a more comprehensive explanation of the role of tRNA dysfunction in nervous system diseases.

Eukaryotic translation initiation factor 4E (eIF4E) phosphorylation by MNK (MAPK-interacting protein kinase) modulates excitatory synaptic activity and depression-like behavior (Amorim et al., 2018). Additionally, eIF4E integrates inputs from ERK (extracellular regulated protein kinases) and mTORC1 (mammalian target of rapamycin complex 1) signaling, and has been shown to influence pain plasticity (Uttam et al., 2018b). eIF2α (eukaryotic translation initiation factor 2α) phosphorylation converts itself into a competitive inhibitor of eIF2B (eukaryotic translation initiation factor 2B), a guanine nucleotide exchange factor, thereby aggravating cognitive deficits after traumatic brain injury (Tsai et al., 2018; Zyryanova et al., 2018). Other translation initiation and elongation factors, including eukaryotic elongation factor 1A (eEF1A), and eukaryotic elongation factor 2 (eEF2), are involved in neurodevelopment and long-term synaptic plasticity (Beckelman et al., 2016; Jan et al., 2018). However, few studies have examined the role of eukaryotic release factors in neurological disorders.

Non-coding RNAs and RNA binding proteins have emerged as key regulators in the nervous system. miRNAs (micro RNAs) modulate protein levels by typically binding to the 3′-untranslated region of cytosolic mRNA targets, triggering mRNA degradation (Germany et al., 2019). miR-134, a brain-specific miRNA negatively modulates synapse development by inhibiting LIM kinase 1 protein translation (Schratt et al., 2006). miR-144-3p represses translation and is linked to the rescue of anxiety-like behavior (Murphy et al., 2017). Mounting evidence indicate that lnc-RNA (long non-coding RNAs) and circRNAs (circular RNAs) act as competing endogenous RNAs by sponging microRNAs in neuronal cells (Zhang H. et al., 2018; Chen et al., 2020). FMRP (fragile X mental retardation protein), an RNA binding protein, appears to directly bind to the 80S ribosome, thereby repressing translation of target mRNAs, and has been linked to autism (Darnell et al., 2011; Chen et al., 2014). The RNA-binding protein, PUM2 (Pumilio2), inhibits translation of genes involved in mitochondrial homeostasis and is induced in the aging brain (D’Amico et al., 2019). Deletion of Lin28, an RNA binding protein, is associated with brain developmental defects (Herrlinger et al., 2019).

Ribosome-nascent chain complex, an intermediate product of translation, will be discussed in detail under narrow translatomics. Detailed analysis of mRNA translation, including translation rates, can provide insight into the regulatory effects of broad translatomic components in the nervous system (Darnell et al., 2011). Collectively, the above evidences show that orderly translation is essential for maintaining neuronal homeostasis and aberrant translational elements contribute to neurological diseases.

## Narrow Sense Translatomics

**FIGURE 2:**
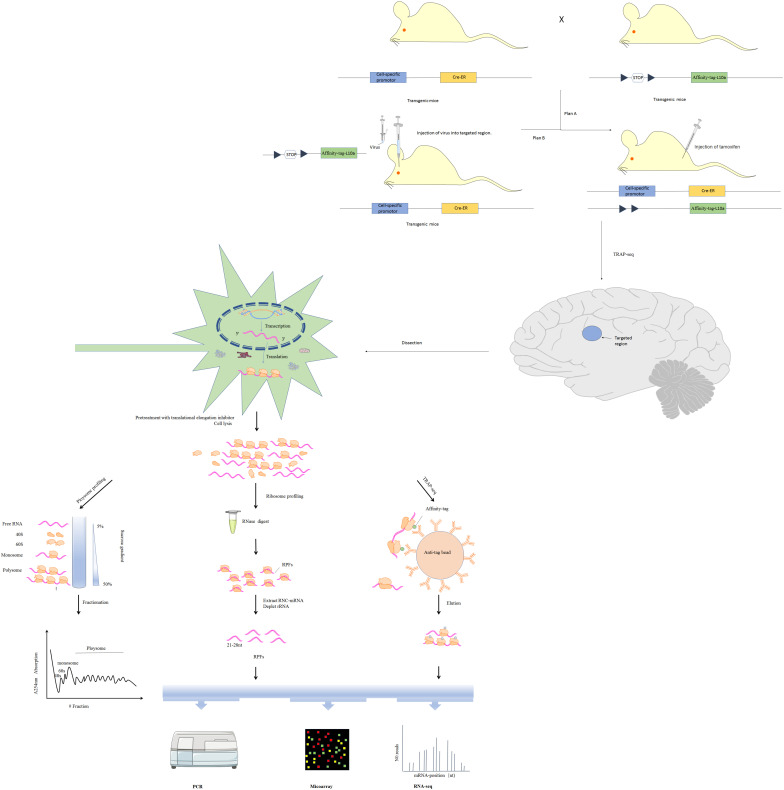
Three major translatomic methods investigating RNC-mRNAs [modified from Figure 1 in Zhao et al. (2019)]. Polysome profiling: utilizing features of the largest ribosome sedimentation coefficient in most cells and separating translating mRNAs with polyribosomes by sucrose density gradient ultracentrifugation and fractionation. Ribosome profiling: treating the ribosome-nascent peptide chain complex with ribonuclease to degrade mRNA fragments that are not covered by ribosomes, and isolating ribosomes protected fractions (RPFs). TRAP-seq: isolating RNC-mRNAs of specific cell type by affinity purification with corresponding anti-tag beads, under the control of a cell-specific promoter and an affinity tag (such as His, Avi, GFP, etc.) which is fused into the large ribosomal subunit (such as Rpl10a protein). The construction of transgenic cre mice expressing Rpl10a protein with affinity tag in specific cell in the nervous system is shown.

In the narrow sense, translatomics refers to investigation translating mRNA in order to elucidate translational changes. Due to non-covalent interaction between ribosomes and mRNA, ribosomal nascent-chain complexes are highly unstable and tend to dissociate during cell lysis, increasing chances of enzymatic degradation (Zhao et al., 2019). Multiple approaches, including microarray-based sequencing and RNA sequencing (RNA-Seq) have been developed for transcriptome identification and quantification (Wang et al., 2009). Based on RNC features and transcriptomics methods, multiple techniques have been developed for analyzing translation, including polysome profiling, ribosome profiling (Ribo-seq) and translating ribosome affinity purification (TRAP-seq) (Figure 2; Zhao et al., 2019). Here, we will review technical advances that have provided insight into translational regulation in nervous system diseases.

### Polysome Profiling

One or more ribosomes recruit identical mRNA with the translation rate being limited by initiation rate. Thus, ribosome density on a given mRNA reflects translational status. Polysome profiling is a technique developed in the 1960s that relies on sucrose density gradient ultracentrifugation and fractionation (Drysdale and Munro, 1967). mRNAs bound to varying numbers of ribosomes can be separated via centrifugation (King and Gerber, 2016). Polysomes and monosomes are often separated through a standard linear sucrose gradient (commonly 5–50% sucrose) made by a gradient maker (Liang et al., 2018). Gradients are collected into several fractions, some of which are translating mRNAs associated with polysomes, monosomes and the supernatant containing the free mRNAs, 60S and 40S ribosomal subunits (Figure 2). The height of polysome peaks of the curve and the area under each peak indicate ribosome translational activity (Figure 2). Northern blot, RT-qPCR, as well as the high-throughput microarray or RNA-seq approaches are then used to identify mRNAs in the separated components (Figure 2). Initiation inhibition causes ribosome “runoff,” leading to decomposition of polysome and elevated levels of free ribosomal subunits. Elongation inhibition enhances polysomal size. Detection of similarly sized polysomes in different circumstances suggests that initiation and elongation have both been affected or translation activity is unchanged (Gandin et al., 2014). The unfeasibility of handling many samples in parallel limits wide use of polysome profiling. Furthermore, it is difficult to distinguish between stalled ribosomes and active polysome whose distribution may change within the high fraction (Sivan et al., 2007). These challenges can be partly surmounted by ribosome profiling (see details below).

### Ribosome Profiling

Previously, there have been no ways to directly quantify protein synthesis rates. Thus, studies of translational regulation have relied on comparing mRNA and protein levels. Although polysome profiling may estimate protein synthesis, this approach has low resolution and accuracy. Ingolia et al. developed the ribosome profiling method in which the ribosome-nascent peptide chain complex is nuclease-treated to destroy mRNA fractions that are not occupied by ribosomes (Figure 2; Ingolia et al., 2009). Ribosome-protected fractions (RPFs) are then isolated by sucrose density gradient centrifugation or immunopurification of ribo-tag cells (Ingolia et al., 2012). After ribosome and rRNA removal, high-throughput sequencing is used to detect small, ribosome-protected, RNA fragments of about 21–28 bp. This approach has multiple advantages. High-resolution ribosome footprints (RFs) enable genome-wide analysis of translation with codon resolution and can: (i) elucidate translational efficiency of all individual mRNAs as calculated from ribosome profiling paired with RNA-seq (Ingolia et al., 2009), (ii) detect even relatively rare and subtle translation events (Brar and Weissman, 2015), (iii) uncover rich and precise ribosome positional information, such as translation initiation at non-AUG codons (Spealman et al., 2018), identify upstream ORFs (uORFs) translation (Rodriguez et al., 2019), as well as elucidate codon usage bias (Paulet et al., 2017) and ribosome pausing (Michel et al., 2012).

The availability of commercial kits has greatly simplified ribosome profiling. However, its efficiency in accurately measuring mRNA translation efficiency is limited by the following factors: (i) independent biases caused by ribonuclease digestion (Gerashchenko and Gladyshev, 2017), (ii) biases introduced by translation elongation inhibitors, for example, anisomycin and cycloheximide favor 21 nt RPFs and 28 nt RPFs, corresponding to ribosomes with open and occupied A sites, respectively (Lareau et al., 2014; Wu et al., 2019), (iii) relatively high false positive and negative rates due to bias toward analysis of main coding sequences (CDS) and lack of rRNA probes for complete rRNA depletion (Guttman et al., 2013; King and Gerber, 2016).

### TRAP-Seq

Isolation of full-length mRNAs associated with ribosomes has been conventionally done via polysome profiling. However, this approach is time-consuming and limited to handling large numbers of samples in parallel. Alternatively, translating ribosome affinity purification sequencing (TRAP-seq) based on expressing tagged ribosomal proteins has been reported (Inada et al., 2002). By using animals or cells in which activated cre-recombinase under a cell-specific promoter drives expression of an affinity tag (such as His, Avi, or GFP) fused to the large ribosomal subunit, RNC-mRNAs of specific cell type may be isolated by affinity purification with corresponding anti-tag beads (Figure 2; Heiman et al., 2014). This approach is limited by the need to generate stably transfected cell lines or transgenic animals for cell type. The potential biases of this method are that some ribosomal proteins including RPL10a preferentially translate specific mRNAs (Xue and Barna, 2012), and that some isolated mRNAs may be extraribosomal.

Translating ribosome affinity purification sequencing is seldomly used in other fields. However, it is especially important in the nervous system because it is relatively difficult to reliably obtain translational descriptions of specific cells from the central nervous system tissues due to its complex cellular heterogeneity (Okada et al., 2011). Although Fluorescence Activated Cell Sorting (FACS) method has been able to isolate specific cells of brain and Schwann cells of the peripheral nervous system (Rubio et al., 2016; Germany et al., 2019; Tomlinson et al., 2020), it takes one whole day to dissociate the tissue and isolate cells by FACS (Rubio et al., 2016). Thus, it may change gene translational expression patterns and limit the utility of time-consuming translatomics methods including polysome profiling and ribosome profiling. In this case, TRAP-seq may be the only practical way to investigate cell-type specific translation for neuron cells and Schwann cells.

### Other Translatomics Method

It is widely accepted that actively translating mRNAs bind multiple ribosomes. However, this is contradicted by recent findings that translation occurs also on mRNAs bound to monosomes, and that translation is not directly proportional to the number of ribosomes on mRNA (Requião et al., 2017; Neidermyer and Whelan, 2019; Biever et al., 2020). Relative to standard linear gradient of polysome profiling, non-linear gradients of RNC-seq (full-length translating mRNA sequencing) enable translating mRNAs elution at smaller volumes to circumvent the limitations of polysome profiling (Liang et al., 2018). In the RNC-seq approach, cell lysate is transferred to the surface of 30% sucrose buffer and the bottom pellet containing RNC-mRNAs collected from the sucrose solution after ultracentrifugation (Liu et al., 2018; Zhang M. et al., 2018). Although high-resolution ribosome profiling defines elongating ribosomes along mRNA, it cannot record intermediates of translation initiation, termination and recycling. To address this, Stuart K. Archer et al. developed translation complex profile sequencing (TCP-seq) method which developed from ribosome profiling (Archer et al., 2016). The yeast cells were crosslink with formaldehyde to stall ribosomes and attach translation complex to mRNA at native positions. Then translation complex subjected to RNase digestion. Next, the ribosome and small subunit (SSU) were separated by sucrose gradient ultracentrifugation, and the 250 nt-long RNA fragments on these fragments were sequenced to obtain the naturally distributed map at the initial, extension and termination stages of translation, respectively. The method is capable of observing the SSU footprint on the 5′ untranslated region (UTR) of mRNA and capturing the position of any type of ribosome -mRNA complex at various stages of translation. For valuable, limited amounts of nervous system tissue, RNC-seq seems superior to polysome profiling. However, RNC-seq and TCP-seq are seldomly used in neurology field, probably because they are relatively new and not known to most neurologists.

### Applications of Translatomics for Neurological Disease

Each method of translatomics has its own unique application and advantages. Polysome profiling, the first translatomics technique, can measure ribosome density and fractionate RNA based on ribosome occupancy. Methods that provide precise mRNA positional information of ribosome occupancy can accurately estimate translation rate. TRAP, which is the most widely used in nervous system studies can analyze translation in specific cell types. To better show the role of translatomics in the nervous system, we focused on the application of polysome profiling in glioma disease, ribosome profiling in FXS and Parkinson’s disease (PD) by virtue of ribosome stalling identification. TRAP in neuropathic pain, depression, multiple sclerosis (MS), and memory consolidation associated with Alzheimer’s disease (AD) through targeting cell types defined by genetics or projections and monitoring local translation.

#### Glioma

In polysome fractionation, the height of polysome peaks and the area under each peak indicate ribosome translational activity. Brain cancer such as Glioblastoma Multiforme (GBM) which is an intrinsic and highly heterogeneous tumor of the brain has been studied by the classic and affordable methodology of polysome profiling due to having a variety of recognized tumor cell lines (Westphal and Lamszus, 2011). Analysis of translational rate, expressed as the ratio of area under the polysome and 80S peaks, and analysis of total and translating mRNAs levels by combining tanslatome and transcriptome methods, revealed for the first time that translating mRNAs can act as biomarkers for glioblastoma and previously unappreciated GBM heterogeneity (Bernabò et al., 2017; Lupinacci et al., 2019).

Glioblastoma stem cells (GSCs) within GBM can self-renew, differentiate into distinct lineages and escape current therapeutic approaches including radiotherapy and chemotherapy (Bayin et al., 2014). Overcoming the escape mechanism could make substantial progress toward an more effective treatment. As a clonogenic subpopulation, the GSC lines are critical to study the treatment response of glioblastomas, and thus polysome profiling with high demand for sample size was widely used. Wahba et al. analyzed radiation-induced translatomes and put forward new molecular insights concerning GBM radiosensitization such as Golgi dispersal and increased eIF4F-cap complex formation detected after radiation (Wahba et al., 2016). Radiation primarily modifies gene expression via translational control (Wahba et al., 2016). Moreover, the nuclear export protein XPO1 in the interacting network based on translatome data was identified to serve as a key molecule for radiosensitization in GSCs (Wahba et al., 2018). Of significance, *in vitro* and *in vivo* experiment proved that the XPO1 inhibitor selinexor enhanced the radiosensitivity of GSCs by inhibiting DNA repair. In addition to radioresistance, drugresistance also needs to be solved. Proneural (PN) and mesenchymal (MES) GSCs are two mutually exclusive GSC populations with distinct dysregulated signaling identified by genome-wide transcriptional analysis (Nakano, 2015). The two subtypes drive therapeutic resistance in glioblastoma. It has been showed that arsenic trioxide (ATO) drug is more potent and inhibitory to PN GBM cells than to MES GBM cells. ATO activates the MAPK-interacting kinase 1 (MNK1)-eukaryotic translation initiation factor 4E (eIF4E) signaling axil, and MNK activation associates with ATO resistance (Bell et al., 2016). Given this information, Bell et al. used polysomal fractionation to analyze an ATO-induced translatome and found an enrichment of anti-apoptotic mRNAs, suggesting translation-mediated resistance to ATO in MES GSCs (Bell et al., 2018). Furthermore, in an apoptosis assay, inhibition of MNK sensitized MES GSCs to ATO (Bell et al., 2018). This finding raises a possibility that targeting MNK1 makes MES GSCs sensitive to drugs such as arsenic trioxide. Collectively polysome profiling gives insight into the radiation and drug resistance of GBM cells at the translation level.

#### Neuropathic Pain

Neuropathic pain is a disorder that features spontaneous persistent or shooting pain after nerve injury. Translational regulation is key in nociceptive circuits and altered mRNA translation is involved in the sensitization of nociceptors in response to nerve injury (Khoutorsky and Price, 2018).

Dorsal root ganglion (DRG) and trigeminal ganglion (TG) neurons are remarkably nociceptive. Which mRNAs translation has been changed in nociceptors causing neuropathic pain is unclear. Translatomics methods can help to solve this problem. The most straightforward application of TRAP is the systematic molecular characterization of target cell types in complex tissues, including the brain, from which neurons are extremely difficult to isolate. Doyle et al. used 16 transgenic mouse lines to compare translational profiling of distinct cell types of the nervous system (Doyle et al., 2008). This versatile strategy could molecularly characterize specific cell types even in the most heterogeneous cell populations *in vivo*. Thus, TRAP complements standard cell sorting approaches that may be influenced by artifacts and bias toward some subtypes (Haimon et al., 2018; Huang et al., 2019). Regardless, droplet-sequencing (Drop-seq), like single-cell approaches, have advanced enough to enable counting of specific cell types (Saunders et al., 2018), and TRAP remains a convenient way of investigating translational regulation.

A recent study used Nav1.8-TRAP mice to achieve sensory neuron-specific ribosome tagging with enrichment in the nociceptor population (Megat et al., 2019a). This unbiased method demonstrated a novel translation regulation signaling circuit causing chemotherapy-induced neuropathic pain (CIPN). Mitogen-activated protein kinase interacting kinase (MNK) and eukaryotic initiation factor (eIF) 4E activity acted on the target of rapamycin complex 1 (mTORC1) via control of RagA translation in Nav1.8-TRAP mice with CIPN (Megat et al., 2019a). In the same mice, they observed higher enrichment in mechanistic target of rapamycin (mTOR)-related genes in the TG-TRAP dataset compared with DRG, whereas translational efficiency in AMP-activated protein kinase related genes was higher in the DGR. Moreover, capsaicin stimulation of the TG region caused greater pain responses than stimulation of DRG-innervated regions (Megat et al., 2019b). This finding is in line with the fact that the mTORC1 signaling pathway plays a key role in controlling nociceptor excitability and sensitization, and this sensitization is strongly weakened by AMPK pathway activation (Schmidt et al., 2016). The several published studies on pain mechanisms and translatomics almost entirely limited to the peripheral nervous system, and the only one targeting the central nervous system was performed by Uttam et al. (2018a), who adopted the ribosome profiling approach to identify mRNAs with significantly changed translational efficiency in spinal cord dorsal horn tissues after spared nerve injury (SNI).

Multiple studies have confirmed that translation also occurs at decentralized local neuronal domains that are highly polarized, such as in the peripheral nervous system (PNS) axonal and central nervous system (CNS) dendritic compartments (Glock et al., 2017). IL-6 and NGF in primary afferent neurons and their axons cause nascent protein synthesis by enhancement of the eIF4F complex formation to induce pain hypersensitivity *in vitro* (Melemedjian et al., 2010). Inhibition of protein synthesis with local administration of rapamycin in myelinated axons of the rodent foot pad was shown to prevent capsaicin-induced hyperalgesia (Jiménez-Díaz et al., 2008). The local translation regulation mechanism has a key role in the establishment and maintenance of chronic pain. Despite evidence indicating that mature axons are capable of protein synthesis (Kalinski et al., 2015; Rangaraju et al., 2017), the small size of axons and tight connections with glia and post-synapses have made *in vivo* transcriptome and translatome isolation extremely challenging. Importantly, microdissection-based approaches are limited to brain regions (e.g., the hippocampus; (Biever et al., 2020) where axons are in separable lamina. While abundant transcriptome analysis demonstrated that the mRNAs for cAMP-response element binding protein (CREB), Na_*v*_1.8, and other ion channels localize to DRG axons *in vitro* (Thakor et al., 2009; Melemedjian et al., 2014; Hirai et al., 2017), there are technological challenges to research in which mRNAs are translated locally in nociceptor axons *in vivo*. TRAP-seq has overcome this limitation. Because the axons of retinal ganglion cells terminate in the superior colliculus of the midbrain, Shigeoka et al. used axon-TRAP, in which the cyclization recombination enzyme is only expressed in retinal ganglion cells, and immunopurified mRNAs on ribosomes in developing and mature axons from the dissected superior colliculus (Shigeoka et al., 2016). Axon-TRAP may expand our understanding of which mRNAs are locally translated to regulate excitability in nociceptor axons.

#### Depression

According to the World Health Organization, at least 350 million people are affected by depression worldwide (Smith, 2014). Depression, one of the psychiatric disorders, has become a growing health concern. Several studies have reported the involvement of astroglial cells in depression, and significant changes in astrocytes in response to antidepressants (Banasr and Duman, 2008; Nagy et al., 2015; Peng et al., 2015). However, the nature of these significant changes remains largely unknown. Simard et al. employed astroglial-specific bacTRAP mice exposed to chronic variable stress (CVS) to generate a translatomic database of differentially expressed genes (DEGs). Analysis of top DEGs indicated that CVS impaired perineuronal net (PNN) degradation, resulting in neuroplastic dysfunction (Simard et al., 2018). Subsequently, based on the same database, Coppola et al. used bioinformatics analysis to further delineate key mediators involved in the astroglial-PNN-depression relationship and found important transcription factors in astroglial cells (Coppola et al., 2019). In addition to PNN, the serotonin system plays a critical role in the pathogenesis of stress, but therapies that target the stress and depression associated genes such as Tph2 and Slc6a4 (SERT) to modulate serotonin reuptake and degradation have no obvious effect in clinical trials (Lalovic and Turecki, 2002; Bellivier et al., 2004; Oquendo et al., 2014). Directly targeting serotonin signaling is expected to be an alternative therapeutic. A study of immunoprecipitated mRNAs associated with ribosome of serotonin neurons in ePet-Cre^*tg/*–^/RiboTag^*tg/*–^mice (Scott et al., 2005) with conservative sequencing analysis showed that *Fkbp5* mRNA translation decreased in the dorsal raphe brain region after repeated forced swims (Lesiak et al., 2020). The results not only support previous findings linking FKBP5 to depression, but also provide the first evidence linking FKBP5 to serotonin. Translatomics connects the three, and FKBP5 inhibitors are shown to have promise as potent and novel antidepressant treatments.

#### Fragile X Syndrome

Fragile X syndrome is a neuro-developmental and monogenic disease associated with autism spectrum disorder (ASD). FXS results from the loss of FMRP encoded by fragile X mental retardation 1 (FMR1). FMRP, a polyribosome-associated RNA binding protein, has always been thought to be involved in translational repression and in the maintenance of tuned protein synthesis, but there has been little consensus regarding the translational mechanism causing FXS before the use of translatomics. A study by Darnell et al. using crosslinking immunoprecipitation (HITS-CLIP) and polysome profiling to assess correspondence between FMRP and mouse brain-specific polyribosomal mRNAs, uncovered its role as a ribosomal brake, and showed that loss of the translational brake contributed to FXS (Darnell et al., 2011). However, stalled and active ribosomes move to the high sucrose concentration fraction during sucrose gradient centrifugation, implying that polysome profiling does not provide a perfect demonstration of translational activity (Sivan et al., 2007). Similarly, the TRAP method immunoprecipitates all ribosome-bound mRNA, and thus cannot differentiate between stalled and active ribosomes. It is also unclear whether FMRP significantly represses both initiation and elongation. Ribosome profiling yields insufficient information on overall ribosome density and ribosome stalling on each gene at high resolution. Supporting evidence has come from studies that measured and compared ribosomal occupancy, positioning, and translation efficiency (TE) in Fmr1 knockout vs. wild-type mice. These studies uncovered widespread decline of translational pausing but no significant translation initiation changes in Fmr1 knockout mice (Das Sharma et al., 2019). Translatomics, especially ribosome profiling, dissects the nature of FMRP-mediated translational regulation. However, ribosome profiling used by Sharma et al. may not distinguish between translocating and stalled ribosomes, suggesting that key events leading to FXS may be overlooked. To circumvent this issue, Shah et al. discovered inhibited elongation in Fmr1 knockout mice by virtue of reduced TE and increased protein level. In order to distinguish between transiting and stalled ribosomes, a modified procedure was used. After blocking initiation of all hippocampal-cortical slices, followed by translocation inhibition at different time points using cycloheximide, ribosome profiling was then used for sample analysis (Shah et al., 2020). This approach detects mRNA-specific ribosome translocation dynamics in the nervous system, which is a remarkable breakthrough.

#### Neurodegenerative Diseases

A range of neurodegenerative diseases such as Parkinson’s disease (PD), Alzheimer’s disease (AD), and multiple sclerosis (MS) are increasingly considered to have molecular mechanisms including protein aggregation (Ross and Poirier, 2004). Parkinson’s disease (PD) is the second most prevalent neurodegenerative disorder, with motor and non-motor symptoms (Samii et al., 2004). The most common genetic cause of Parkinson’s disease is mutations in the *LRRK2* gene. Kim et al. estimated translation efficiency by comparing between the ribosome-profiling and the RNA-seq expression changes, and translational landscape demonstrated a global shift in G2019S LRRK2 human dopamine neurons (Kim et al., 2020). Of note, differentially regulated genes had a common feature with complex secondary structure in the 5′ untranslated region (UTR), and Ingenuity Pathway Analysis (IPA) showed Ca^2+^ signaling, which indicated elevated intracellular calcium levels (Kim et al., 2020). Ribosome profiling associated dysregulated translation control with intracellular Ca^2+^ homeostasis imbalance in G2019S LRRK2 human dopamine neurons. Alzheimer’s disease (AD) is the leading cause of dementia, characterized by regression of learning and memory. Thus, it is necessary to study the translational regulatory mechanisms that affect memory to inhibit the onset and progression of AD. Although the importance of translational regulators in memory consolidation has been shown, our understanding of target genes under translational control in memory formation is limited by the lack of accurate ways of quantifying translation rate (Costa-Mattioli et al., 2005). Cho et al. analyzed translation efficiency through temporal ribosome profiling and transcriptome profiling of the mouse hippocampus during memory consolidation and identified 3 different types of translationally repressive gene regulations distributed in steady-state, early, and late-phase (Cho et al., 2015). However, Mathew et al. pointed out that the probable inclusion of choroid plexus mRNA in hippocampal samples used by Cho et al. needs to be considered before drawing conclusions. Regional differences may occur in the transcriptome of each cell type during neurodegeneration. Thus, isolation of neurons from entire brain tissue needs to be resolved (Cho et al., 2016; Mathew et al., 2016). Cre-LoxP recombination can successfully generate ribo-tag mice expressing tagged ribosomes in specific cell types. Using *Cx3cr1*^*CreER/CreER*^; *Rosa26*^*fsTRAP/fsTRAP*^ mice, it was found that the chronic environmental risk factor Cu exposure impaired cognition and increased the incidence of AD by shifting microglia toward inflammatory phenotypes at translational levels (Lim et al., 2020). For multiple sclerosis (MS), another neurological degenerative disease, Olig1-RiboTag mice permit isolation of oligodendrocyte lineage cells, and allow specific translatomics *in vivo* from targeted regions during the remyelination phase of a MS model, and discovery of gene-expression pathways intrinsic to oligodendrocytes with MS (Voskuhl et al., 2019). Accurately interfering with the translation mechanism in specific cells of targeted regions may be novel therapeutic targets for neurodegenerative diseases.

The brain contains a diverse population of projection neurons with anatomical and molecular heterogeneity. Dissociating each projection neuron by laser-capture microdissection leads to the loss of RNA pools located in dendrites and axons, which account for >50% of the neuronal volume (Tóth et al., 2018). Furthermore, cell isolation protocols are relatively inefficient, while TRAP has sufficient power to simultaneously analyze hundreds of cells for a candidate cell type (Huang et al., 2019). Although ribo-tag mice constructed with cre-loxp recombination have promoted the investigation of translation regulation on memory consolidation in cell types of specific brain regions, more specific spatial translational regulation such as axon-translation achieved by viral-TRAP could provide new insights into memory formation in projection neurons. Ostroff et al. injected retrograde virus into cortical area TE3 to express tagged Rpl10a (ribosomal protein L10a) at axon terminals, resulting in tagged Rpl10a expression in retrogradely transduced projection neurons of the amygdala region (Ostroff et al., 2019). Combined with RNA-seq, genome-wide biomolecular profiling of anatomically defined projective neurons can be performed. Ostroff et al. using viral-TRAP not only proved the recent assumption that axonal translation also occurs in the CNS neurons *in vivo*, but also demonstrated that axonal translation in projection regions is associated with memory formation. In addition to these viruses, which do not cross synapses, transsynaptic viruses may trace inputs farther upstream. Canine adenovirus type 2 – green fluorescent protein (CAV – GFP), a transsynaptic virus, was injected into particular brain regions including the nucleus accumbens shell, of transgenic mice whose ribosomal proteins fused with anti – GFP camelid nanobody, and was used to molecularly profile neurons projecting to farther regions (Ekstrand et al., 2014).

### Potential Applications of Translatomics in Neurological Diseases

#### Alternative Transcript Isoforms

The nervous system exhibits the most divergent and extensive use of alternative transcription isoforms, which may influence development of genetically-defined neuron types, neuronal maturation, and synapse specification (Furlanis and Scheiffele, 2018). Splicing misregulation is involved in the development and maintenance of neurologic diseases (Licatalosi and Darnell, 2006). High-throughput RNA-seq of autistic brains has identified a large number of downregulated alternative splicing of neuronal activity-dependent exons (Parikshak et al., 2016; Quesnel-Vallières et al., 2016). However, it is not clear to what extent transcription isoforms detected by RNA-seq are indeed translated into functional protein isoforms (Weatheritt et al., 2016; Tress et al., 2017). In addition to arising from transcription isoforms, protein isoforms may arise from same transcript encoding multiple protein variants via alternative translation initiation sites and termination sites. Although translatomics has not been used to explore whether the process of transcription isoforms to protein isoforms also plays an important role in neurological diseases such as autism, the possible mechanism of protein isoform production by translatomics in nervous cells has been studied (see below). This could lead to further study of diverse protein isoforms in neurological diseases.

As opposed to ribosome profiling in which only the coding sequence protected by ribosomes is detected, polysome profiling and TRAP can detect full-length translated mRNAs, including untranslated regions. Wong et al. used polysome profiling to investigate differential translation rates as quantified by shifts in ribosomal load between variant mRNA isoforms with alternate 5′ UTRs in embryonic stem cells-derived neural precursor cells (Wong et al., 2016). Furlanis et al. used TRAP to probe transcript isoforms that are recruited for translation across distinct neuron types (Furlanis et al., 2019). TRAP minimizes low-level background noise from widely expressed non-neuronal genes and facilitates detection of divergent alternative splicing programs across closely-related cell types. These two methods offer new possibilities to study contexts in which transcript isoforms are not completely translated into alternative splicing proteins. Ribosome profiling with codon resolution was used by Sapkota et al. to uncover novel translation initiation sites in the same transcript, which give rise to N-terminal protein variants, and to identify new C-terminal extensions mediated by stop codon readthrough in neuron-glia cultures (Sapkota et al., 2019). Moreover, this study coupled TRAP to ribosome footprints for specific cell type analysis of translational readthrough *in vivo*. Taken together, the three translatomics methods have greatly enhanced our knowledge on diversity of protein isoforms.

#### Novel Neuropeptides

As signaling molecules and key mediators, brain-derived peptides are responsible for brain activities, including learning, memory, and stress (Borbély et al., 2013; Fazzari et al., 2018). Most reported neuropeptides to date are characterized as ligands for G-protein coupled receptors (GPCRs), which serve as extraordinarily efficient drug targets in general (O’Brien et al., 2019; Li et al., 2020). Given their therapeutic potential, significant efforts have been made to search for novel functional peptides in basic neuroscience. Currently, micropeptides from the brain are receiving more attention in translational neuroscience. However, the low abundance and atypical fragmentation of brain-derived peptides limits the effectiveness of conventional proteomics. The position of ribosomes and protected footprints by ribosome profiling facilitate identification of translation products and discovery of novel protein products. The combination of ribosome profiling and mass spectrometry may facilitate their detection in the brain (Tharakan et al., 2020). Moreover, the ribosome footprint distribution can identify translated upstream open reading frames (uORFs) (Jiang et al., 2017) and novel translation initiation or termination sites (Sapkota et al., 2019), providing clues for the discovery of novel micropeptides.

#### Single mRNA Molecule Translation

While the above-mentioned translatomics methods including polysome profiling, ribosomal profiling, and TRAP-seq have provided quantitative new insights for translation regulation *in vivo*, genome-wide methods require averaging of many mRNA molecules in cells, ignoring differences between individual mRNAs. To visualize translation dynamics of single mRNAs in living cells, Halstead et al. first developed a technique called translating RNA imaging by coat protein knock-off (TRICK) (Halstead et al., 2015), in which orthogonal bacteriophage PP7 and MS2 stem-loops were used to label a transcript with distinct fluorescent proteins. The PP7 capsid protein was fused with green fluorescent protein (PCP-GFP), and the MS2 capsid protein was fused to red fluorescent protein (MCP-RFP). Untranslated mRNAs simultaneously express green and red fluorescent proteins, while in contrast translated mRNAs which are at least undergoing the first round of translation are only labeled with MCP-RFP in the 3′ UTR. However, the TRICK technique is more suitable for detecting the first round of mRNA translation in living cells. A method based on the recently developed SunTag fluorescence labeling system to label nascent polypeptides of mRNA was described (Morisaki et al., 2016; Wang et al., 2016; Wu et al., 2016; Yan et al., 2016). In this method, the MS2 and PP7 systems (Halstead et al., 2015) are used to image mRNA to obtain the quantitative and positional relationship between mRNAs and the ribosome. Only one labeled polypeptide can be detected when using a fluorescence labeling system alone. Boersma et al. applied two independent MoonTag and SunTag systems with different fluorescent proteins, so the spatial-temporal translation of two different mRNAs can be observed in living cells (Boersma et al., 2019). Furthermore, the combined MoonTag and SunTag systems can image stop codon readthrough of single mRNAs by putting MoonTag in the open reading frame of the gene and SunTag after the stop codon (Boersma et al., 2019).

Few studies have achieved image translation of single mRNA molecules in living neurons. These found that translation was repressed in distal dendrites of primary neurons because ∼40% mRNAs were translated in proximal dendrites, but only ∼10% mRNAs were translated in distal dendrites (Wang et al., 2016; Wu et al., 2016). The bursting translation behavior of these mRNAs can effectively control the level and location of protein in synapses. This method is thus very important to explore the pathogenesis of mental disorders with synapse impairments such as fragile X chromosome syndrome (Blanco-Suárez et al., 2017). More experiments may be needed to verify and study the translation changes of a single mRNA molecule in nervous system diseases.

### Applications of Translatomics in Other Fields

Translating ribosome affinity purification has many applications in the nervous system with multiple cells. In the nervous system cells, gene translation plays a prominent role in synaptic function (Sossin and Costa-Mattioli, 2019). Polysome profiling, a traditional method for analyzing RNC-mRNAs, can be traced back to the last century. Numerous studies have explored the regulation of gene translation by polysome profiling *in vivo*. Of note, in addition to obtaining RNC-mRNAs that has been following, polysome profiling and TRAP can also be utilized to isolate ribosome-association protein complexes that are immunoprecipitated from gradient fractions or purified polysome-associated mRNA transcripts. The physiological functions of such protein complexes can be determined by western blotting or mass-spectrometry (Gandin et al., 2013; Simsek et al., 2017). One shortcoming of polysome profiling is that the slow gradient fraction and translation ribosome affinity purification method results in dissociation of tightly bound proteins and hence does not isolate proteins which interact transiently with ribosomes.

**FIGURE 3:**
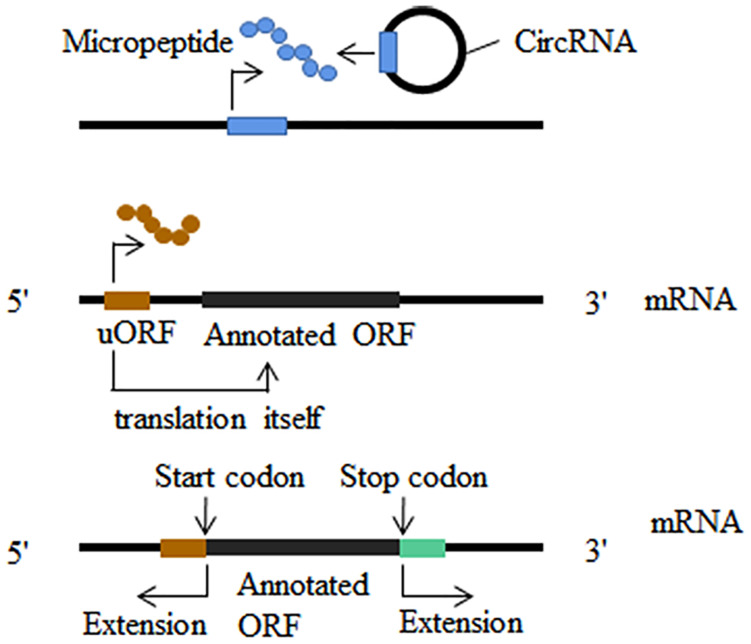
Translatome analysis in other fields. New translatable molecule: non-coding RNAs are found to contain short open reading frames (sORF); upstream open reading frames (uORF) could translate into micropeptides and this translation can influence the translation of the downstream main open reading frames; stop-codon read-through and alternative translation extends in both 3′ and 5′ direction.

Initiation and advancement of translatomics guided by ribosome profiling revealed that some putative long non-coding RNAs (lncRNA) (Lu et al., 2019), microRNAs (Lauressergues et al., 2015) and circular RNAs (Ye et al., 2019) were misannotated as non-coding RNAs. Short open reading frames (sORF) contained in non-coding RNAs are ignored because do their small size (Figure 3). Micropeptides and proteins encoded by non-coding RNAs have been implicated in a variety of biological processes, such as embryonic development (Pauli et al., 2015), muscle function (Anderson et al., 2015), glioma cell suppression (Zhang M. et al., 2018; Wang J. et al., 2019), cell division (Handler et al., 2008) and morphogenesis (Kondo et al., 2007). In addition to non-coding RNAs, new translation patterns related to known coding genes have attracted much research attention. A significant portion of ribosome footprints outside of typical protein coding regions (CDSs) derive from the sequence of 5 ’UTR (Brar et al., 2012; Archer et al., 2016; Murat et al., 2018). Both upstream translation itself and micropeptides encoded by uORF often result in the suppression of the translation of the downstream main open reading frame (Figure 3; Mueller and Hinnebusch, 1986; Vattem and Wek, 2004; Rahmani et al., 2009; Wethmar, 2014; Ebina et al., 2015). Another translation pattern is stop-codon read-through which we have described in the nervous system (Figure 3; von der Haar and Tuite, 2007; Jungreis et al., 2016; Li and Zhang, 2019). Ribosome profiling can also analyze frameshift mutations in the main open reading frame, although it has not been remarkably successful (Atkins et al., 2017).

In addition to the combined analysis with classical omics including transcriptomic and proteomics in the nervous system (Das Sharma et al., 2019; Lupinacci et al., 2019; Wang X. et al., 2019; Tharakan et al., 2020), translatomics can be used in conjunction with m6A-seq to characterize the effect of m6A modification located in the coding region of mRNA on the translation efficiency (Jia et al., 2019; Mao et al., 2019).

## Conclusion and Perspectives

The translation of mRNA into protein is an important cellular process. Any significant changes in the translation components can profoundly affect the development of the nervous system, thereby causing neurological diseases. Translatomics methods have revolutionized our ability to monitor RNC-mRNAs *in vivo* and analyze the regulation of translation. The applications of polysome profiling, ribosome profiling, and TRAP-seq in neurological diseases are listed in Table 1. However, it is clear that better protocols with improved performance are needed for efficient analysis of small samples of nervous system specimens. Compared with the other two methods, ribosome profiling has more gaps in the nervous system. In the future, depending on the precise genomic positional information obtained by ribosome profiling, the stop-codon readthrough occurring in neurons or in glial cells can be used to explore modulating effects of micropeptides encoded by putative non-coding RNAs and uORF on the development of the nervous system. Notably, the method of observing translation of single mRNA molecules in live neurons is a powerful tool for study of translation regulation in neurological diseases. Various combinations of translatomics methods reflecting different aspects of translatome and new translatomics approaches such as RNC-seq and TCP-seq would be useful in neurology field. Application of different omics data types can elucidate potential causative changes that lead to neurological disorders, which is essential for the design of precise and personalized medicine (Hasin et al., 2017). The translatome, an upgraded version of transcriptome, which couples with other omics such as genomics, metabolomics and microbiomics, is likely to promote development of translatomics in the study of the nervous system.

## Author Contributions

SZ conceived and wrote the manuscript. YC modified and reviewed the manuscript. YW, GC, PZ, and YZ edited the manuscript. All authors contributed to the article and approved the submitted version.

## Conflict of Interest

The authors declare that the research was conducted in the absence of any commercial or financial relationships that could be construed as a potential conflict of interest.
